# Nature and Treatment of Decay of the Teeth

**Published:** 1867-08

**Authors:** H. R. Noel


### ARTICLE III.

### *Nature and Treatment of Decay of the Teeth.*

### A Review of Dr. Arthur's Monograph on Decay of the Teeth. By Professor H. R. Noel.

(Continued from page 123.)

There can be no doubt that a temporizing, vacillating procrastinating, course because of the mere age of the child is to be deprecated, and will eventuate in serious mischief. But even the most careful daily attention to the teeth, does not always save them.

"The teeth, are sometimes so defective in structure, or the secretions of the mouth are so acid in character, tnat their presence during the night will gradually destroy the enamel at the point of contact, although the greatest care for their removal should be taken during the day time.—(Page 24.)

Here a question suggests itself as to the degree of influence, which could be excited by proper hygiene and proper therapeutical agents.

We commence with defect in the original conformation of the teeth; defect belonging intrinsically to their intimate structure and composition; also depraved or vitiated secretions about the mouth, both probably dependent upon some vice of the system, hereditary or acquired. To change the nature of the teeth, to alter their intimate structure after complete developement, is not in our opinion, possible. And we believe that the exhibition of minerals, such as lime, magnesia and other earths, combined with phosphoric and carbonic acid, &c., for their direct effect or action upon the teeth, is to say the least, a purely theoretical assumption founded upon the supposed analogy between teeth and bone. This analogy we think to be questionable in the extreme.

As regards depraved secretions, &c., our power is not so limited, Proper remedial agents addressed locally and to the system generally, may restore to a great extent the tone of the secretions, and also the tone and vigor of the constitution. We could scarcely over-estimate the value of health at any age; but its value cannot be estimated, while the developing process is progressing; while the whole system of the child is being formed, being acted upon for good or evil by every agent and influence bearing the slightest relation to disease or health, and the susceptibility to impressions is more keenly alive than ever afterwards.

The foundations of the future are now being laid, and we should see well to the material of which they are to be composed. We cannot too jealously guard the young, and this is especially true of the Digestive system.

The teeth, secretions of the mouth, secretions of the alimentary canal, action of glandular organs, should be most sedulously cared for, This is true! none deny it; and the medical practitioner is required to correct at once, any abnormal action in the digestive tract below the mouth; while too often, the mouth itself, the vestibule, the means of entrance, receives no attention whatever for years, unless severe fever or fetor of breath compel it, and when placed in the hands of the Dentist, presents the sad, crumbling wreck, so often seen; so bitterly repented of when too late.

Is this wise? Is this child receiving even meagre justice? We have examined the mouths of and elicited facts from about 180 persons, within 10 days and the results have been astounding.

In the number 180—taken indiscriminately from all ages, over 12—all occupations, both sexes and both colors, and their modifications, we have found 2 persons under 20 years of age, who have no perceptible decay of the teeth; the rest without exception have decay. Two only in 180—1-90th and this we believe is not exactly correct, and think a more extended observation would have given 1-100th or 1-200th.

The one indisputable fact remains; the teeth of the present generation (from whatever cause it matters not, the fact is the same,) are a grand failure. The theoretical value of enamel finds no corresponding application in its practical; for enamel has failed, utterly and completely failed to realize anything like its theoretical value. We know this statement will meet with opposition, but we are dealing with facts, facts obtained from examination of men individually and in mass, not only by ourselves but by many others, and we assert the truth of the position. The evidence in daily experience even, is overwhelmingly in support of the fact, that enamel as regards its practical value, has been greatly over-estimated. What objection can there be to its removal, when teeth approximate, since the experience of the past and observation of the present, alike demonstrate, that if not removed by the instrument it will be by caries?

The author's leading idea in the monograph hinges upon this fact. The truth is that enamel has failed to give adequate security and protection, and he advocates in the strongest terms the use of instruments wherever the enamel is defective, fissured, &c.; or where teeth approximate in persons predisposed to caries. Smooth surfaces and open spaces are the ends to be obtained. We propose now to make free and lengthy quotations from the monograph.

"It is indeed true, that if the enamel is removed, and the teeth left in the same condition as before its removal, that is, if the affected surfaces be allowed to come again in close contact, decay will recur and go on more rapidly than before the removal of the decomposed parts. But when decay has not penetrated the dentine, or has extended to a very slight depth only,—in this part of the tooth and is removed as above directed, the disease is effectually arrested."—(page 27.)

The signs of decay by means of diagrams, &c., we omit, as being familiar to our readers. Teeth in contact are thoroughly examined, if decayed, separated permanently; if largely decayed, they are filled; if small superficial spots of decay exists they are removed. Teeth should be permanently separated wide enough to admit the bristles of a brush, that they may be kept cleaned. This is very essential, and thorough cleansing should be performed at least twice a day. All particles of food are removed, and the mouth kept as free as possible from deleterious agents. Our author approves pressing teeth apart, filing and then permitting them to fall back again as they were before being operated upon.

"The teeth so treated are then allowed to fall back in their former positions. But it must be seen that unless the decay is of such a character, that the gold alone of the fillings touch, when the teeth come again in contact (which is rarely the case) the condition of the surfaces so far as relates to the circumstances favoring decay, is the same as before the occurrence of the decay; it is worse indeed, for the friction between the gold and the orifice of the cavity can not be so perfect as the surface presented by the intact enamel."—(Page 31.)

The author separates these teeth permanently, whether filled or not.

"After the decay is entirely removed, the surfaces treated should be polished until they present an appearance as vitreous as enamel itself."—(Page 32.)

"Experience has established the fact, that teeth may be deprived of enamel on both sides, and remain free from decay during a long life-time."

"The writer has seen a number of cases, where teeth, one third of which were filled away, remain perfectly sound for the time stated."
Filed by Dr. H. H. Hayden 40 or 50 years ago.—(Page 33.)

"Where the enamel merely is decomposed, or before the dentine is to any great extent, involved, the affected part may be cut away without pain of consequence, commonly with none whatever."

"But when the decay reaches the dentine and penetrates to a slight depth the sensitiveness of the part is greatly increased."—(Page 34.)

"Experience has established the fact, that decay is not liable to occur where space exists between the teeth naturally, or has been artificially made. If there is reasonable certainty that all the teeth, back of the incisors will decay, is it not wise to separate them before decay occurs, as the object is then effected with less loss of substance of the teeth so treated?"—(Page 34.)

"It has been demonstrated that if the decay be removed at an early period after it occurs, and permanent spaces left between the teeth, where it has made its appearance, it is effectually arrested." "If the teeth are separated before it occurs at all, they cannot be in any worse condition than they are, when it is done after decay attacks them."

"The author therefore advises and does not hesitate to put into practice what he advises, the separation of all teeth back of the eye teeth if the incisors are decayed before the 12th year."

We have quoted freely from this chapter, and would suggest a candid investigation of the arguments, facts, &c. Facts are superior to theory

Several deductions can here be made:

1st. Enamel is not absolutely protective.

2d. Its removal does not necessitate caries.

3d. Separation of teeth opposes caries.

4th. Plain, smooth surfaces oppose caries.

5th. High tone of health opposes caries.

6th. Careful attention to the mouth opposes caries.

7th. Filing and separating in early life give little pain.

8th. Filing does not predispose to caries, but rather prevents, by giving open spaces, and a better chance for inspection.

9th. Children are especially adapted to this treatment.

Chapter.—IV. Is a review of the preceeding ground, many cases and examples cited, diagrams given, &c., with the author's experience and conclusions condensed from a careful examination of records kept for the last twenty years.

For fourteen years every important case is fully retained. The method of keeping these records is an admirable one; it is this: upon each page, at the top, are accurate engravings of the upper and lower teeth, so arranged that you look down obliquely upon their cutting surfaces and also upon their internal and posterior aspects; again others, giving the anterior and external aspects of the teeth. As any case is operated upon the work performed is noted down and the tooth marked accurately to show exactly the kind and nature of the labour bestowed upon it. Extracting, filing, filling, &c., have each its distinct and invariable sign or mark, so that with the engravings above and record below, the date of work, character, amount &c., are permanently secured. Comparisons between these engravings and the teeth of the patients, the condition when operated upon and the condition of the mouth after the lapse of months and years, have furnished concise and clear statistical data, upon which the author founds his treatment. Rigid and exact analyses of these cases, as there recorded, have led the author to conclusions not generally acknowledged by the profession. But yet the results are such, as to challenge inspection and study, and make us pause and weigh well the evidence, ere we condemn too hastily from theory, what practical experience seems to assert.

Omitting the numerous cases cited, we will give quotations embracing the results and conclusions arrived at by the author, using the aggregate numbers as we proceed. One book of Record—316 cases—63 transient and not noted; 253 accurately noted—gave 216 cases with more or less decay upon nearly all the sides of bicuspids and molars, at points of approximation; as a rule approximating sides were nearly invariably destroyed; 37 cases had no such decay, but their subsequent history was not obtained.—(Page 50.)

"Taking the first twenty pages of the book referred to, one case recorded on each page, there are found but three in which decay had not occurred, at nearly every point where the teeth are in contact, except the lower incisors."

"Thirteen out of the 17 patients were under 24 and 5 were not above 16 years of age."

"An examination of the other books of record, in the author's possession or of any portion of them shows the same condition of the teeth."—(Page 51.)

Now watch the conclusion at which he arrives, and the logical method with whieh he almost forces the reader to acknowledge the correctness of his treatment.

"If then it be true that in the great majority of individuals the whole ot the teeth back of the incisors, are reasonably certain in time, to be attacked with decay, at or near the points of contact; if these latter show so great a tendency to decay, as to be attacked at a very early age, what is the best treatment for their preservation."—(Pages 51 and 52.)

If the teeth by contiguity, lodge the food, secretions, &c., and thus give the agents acting a longer and better chance to corrode the enamel, they must be permanently separated. The author does not let us stop here, he wishes to prove that time is valuable and children perhaps the best subject for this treatment.

"Now if it is certain that decay will occur ultimately on all surfaces of the teeth, back of the canine teeth, what possible advantage can accrue to the patient from waiting until it becomes *visible extremely?*"

"To wait indeed, until this period of its progress, renders the application of this remedy in most cases impossible."—(Pages 57 and 58.)

"The sides of the molar and bicuspids are so broad, and the decay usually commences so near the gum, that when the first slight discoloration is discovered, by a visual examination, near the grinding surface it will generally, be found that a large cavity has been formed."

"If some exceptional cases, teeth which might never decay, should happen to be separated, in this way, there is no serious injury done, for the teeth so treated are not more liable to decay, than if they had never been touched."

"All the author's experience justifies him in coming *most positively to this conclusion.*"—(Page 58.)

Should decay occur, which in cases of very strong predisposition to, the author's treatment could not prevent, yet even then:

"The surfaces are so exposed (to view) that the slightest attack can be detected."

Here the author brings in many examples to prove his position of "no injury from filing, when well done and a polished surface left." One case, accompanied by an engraving, represents the front teeth of a gentleman 65 years of age, who was operated upon by Dr. Hayden, when he was a mere youth; the Dr. filing two of his teeth for decay and the marks of the file are still there, perfectly distinct but no evidence of a return of the decay thus removed.

This is rather a crucial experiment as regards time.

After citing several cases of children the author sums up as follows:

"A much smaller number of fillings were required to preserve the teeth; the arrest of the decay was effected by a simpler and much less expensive process than filling; it was almost devoid of pain."

These are strong points and we give them great weight; in regard to children this is eminently true and important if true, and we accept the truth of it. Let any one of the profession who doubts its truth, refute it; we would be very grateful if this is not true, for a refutation of it.

Unsightly or disfiguring separations need never be made, unless the teeth are badly decayed, and then it is only a question of time when the whole tooth goes unless filled. Seen in time the operations are nice and appearance good; seen too late it is the fault not of the operation, but the condition of the patient's teeth.

This record book gave, 4966 points of decay, 2298 upon grinding surfaces, results of defective enamel, necessarily filled; 2,668 cases occurred on the sides or surfaces in contact, where the enamel should have been, and doubtlessly was originally perfect. Had the teeth been kept thoroughly clean, no decay need have occurred except in some very bad predispositions to, but the age, &c., of the patient precluded the idea. From various causes the author did not, at that time as rigidly enforce his practice, as he now does, and as a consequence, 1938 fillings had to be inserted; leaving 730 points of decay. Upon these the author remarks:

The decay was removed from 730 places, and the teeth so treated, many of them as long, since as seven years, now remain as perfectly sound as when the operations were performed."—(Page 69.)

The monograph concludes with some remarks upon the rapid increase of decay, and the necessity of some means for arresting it in the early years of life.

Removal of Decay—permanent separation—polished surfaces—constitute the author's treatment in by far the majority of cases of children, when seen in time.

Separation in time constitutes the

"PROPHYLACTIC TREATMENT."

We give this to the profession, as we receive it from the author, at the same time we know many will oppose this practice.

We ask, "prove the error, when you condemn."

The clear, forcible, reasoning of the author; his tenacious earnestness; the inevitable logic of his conclusions if you grant his premises; the stubborn array of facts, and his long practical experience demand of us a cool, unbiassed thoughtful consideration of the subject.

Should he be wrong, he is intensely, enthusiastically wrong; and his position and standing are such that he can do incalculable injury. Should he be right, he is equally as intensely and enthusiastically right; his position upon this question—his views—his opinions, the results of his practical experience, cannot be too widely known among the profession, or too thoroughly and readily adopted. If it be a fact, it is one of the utmost importance, and the profession should know it, and act upon it; if it be not a fact, try it, test it, one year will send it to the winds. Try it by actual, patient experience, discard prejudice and preconceived opinion unless well founded.

Give it a thorough trial, the children of your patrons deserve the experience you will gain by it.

We will accept no theorizing in this matter, unless in accordance with facts; we have given the profession opinions and we have given facts also, and in return our position can only be assailed by opinions founded upon facts. We care nothing for theories, we must have the results of practical experience and patient observation of the phenomena of decay as seen in the living subject.

If the position be a false one, refute it by facts.

The summary would give the following as points for candid consideration.

1. That our best authorities sanction filing as one means of treating decay.

2. That filing does not, when well performed, cause decay, but rather opposes it.

3. Removing the enamel itself does not necessitate decay.

4. That where teeth approximate, decay is most liable.

5. That if the contigous surfaces of the upper incisors show evidence of decay in youth, the prognosis as regards the other teeth at points of contact, is unfavorable.

6. Experience demonstrates the fact, that in by far the majority of persons, teeth in contact sooner or later decay

7. That a permanent separation of the teeth in contact, where a pre disposition to decay exists, even where both enamel and dentine are removed to a limited extent, not only does not increase but greatly diminishes the tendency to decay.

8. Where teeth are separated permanently, they can be better exami ned and decay sooner detected.

9. That this is the true treatment in cases of children; being less painful, less expensive, and more certain than filling.

10. That in all cases, a smooth, plane, highly polished surface should be left, if possible.

In the monograph and in the review of it, the whole question has been one of fact and experience, there is no theorizing about the minute anatomy, histology, &c.; no discussion of theories as to the developeopement of the teeth.

We propose in a future article to examine minutely the opinions of Huxley, Beale and others, as to the structure of the teeth, their developements; their amount of vitality; the question of vascularity, &c., and endeavor to ascertain the true position of enamel and dentine, and then discuss the essential nature of Caries.

The vital theory of Tomes, and the acid theory, will each in turn be subjected to a rigid examination.

We will endeavor to give a physiological explanation of the practica experience, which has lead one author to the

"PROPHYLACTIC TREATMENT OF CARIES."

The equation or problem is this

Given—or known Quantity,

"RESULTS OF PRACTICAL EXPERIENCE."

Wanted—or Unknown Quantity,

"PHYSIOLOGICAL EXPLANATOIN."

